# Follow-up management and the management of high versus low risk patients in primary care

**DOI:** 10.1186/2045-7022-1-S1-S62

**Published:** 2011-08-12

**Authors:** Bertine Flokstra

**Affiliations:** 1 University Medical Center Groningen, Department of General Practice, Groningen, Netherlands

## 

The main aim of this presentation is to give an overview on the follow-up management of food anaphylactic patients and to address the question of which patients should be prescribed an epinephrine auto-injector (EAI) in primary care. Practical recommendations on these issues will be discussed.

A short introduction will be given on the definition of anaphylaxis and the prevalence and incidence of food anaphylaxis in primary care. Although definitions of anaphylaxis do exist, it is not always easy to identify anaphylaxis because the symptoms may be nonspecific and variable. However, it has been shown that the incidence and prevalence of anaphylaxis has increased in primary care.

Once a patient has experienced an anaphylactic reaction, follow-up management is very important. These patients should be referred to an allergy specialist for further evaluation of the food allergy. Meanwhile, these patients should be prescribed an EAI and receive information on how and when to use it. If the culprit food allergen is know, the patient should receive information about avoidance of this food.

In addition, the primary care physician may see other food allergic patients as well who have not (yet) experienced an anaphylactic reaction. In order to decide which of these food allergic patients should be prescribed an epinephrine auto-injector (EAI), an estimation of the risk for anaphylaxis should be made. By taking into account risk factors for anaphylaxis, patients can be divided into low and high risk patients, which will determine further management strategies.

